# Evaluation of the Bio-Rad Geenius HIV 1/2 Assay as Part of a Confirmatory HIV Testing Strategy for Quebec, Canada: Comparison with Western Blot and Inno-Lia Assays

**DOI:** 10.1128/JCM.01398-18

**Published:** 2019-05-24

**Authors:** Bouchra Serhir, Céline Desjardins, Florence Doualla-Bell, Marc Simard, Cécile Tremblay, Jean Longtin

**Affiliations:** a Laboratoire de Santé Publique du Québec/Institut National de Santé Publique du Québec, Sainte-Anne-de-Bellevue, Québec, Canada; b Unité de Surveillance des Maladies Chroniques et de Leur Déterminants/Institut National de Santé Publique du Québec, Québec, Canada; c Département de Microbiologie, Infectiologie et Immunologie, Faculté de Médecine, Université de Montréal, Québec, Canada; d Centre de Recherche en Infectiologie, Université Laval, Québec, Canada; Memorial Sloan Kettering Cancer Center

**Keywords:** Geenius, HIV, cadaveric, confirmation

## Abstract

The rapid confirmatory Bio-Rad Geenius HIV 1/2 assay was evaluated as an alternative to the HIV-1 Western blot (WB) confirmatory assay. A total of 370 retrospective samples collected from 356 patients were tested. Sensitivity of the Geenius assay to detect HIV-1 and HIV-2 infections was 100% and 97%, respectively, and that of the WB assay was 86% and 39%, respectively. Geenius reduced the number of indeterminate results by 85% and exhibited a differentiation capacity for HIV-1 and HIV-2 of 100% and 89%, respectively. Three of 10 patients presenting with an early HIV infection (1 to 2 weeks before seroconversion by WB) were positive using Geenius. None of the HIV-negative samples were positive using Geenius or WB. However, 7% and 10% of them were indeterminate with Geenius and WB, respectively, leading to a specificity rate of 93% for Geenius and 90% for WB. Ninety cadaveric samples (54 negative, 23 HIV-1 positive, and 3 HIV-1 indeterminate) were tested with Geenius, leading to a sensitivity of 100%, a specificity of 96%, and an indeterminate rate of 4%. Our results indicate that the Bio-Rad Geenius HIV 1/2 rapid test exhibits better sensitivity to detect HIV-1 infections and better performance than WB to confirm and differentiate between HIV-1 and HIV-2 infections. The performance of this new confirmatory assay to detect early infections, to reduce the rate of indeterminate status, and to confirm HIV-1 infection in cadaveric blood samples makes Geenius a potent reliable alternative to the WB.

## Abstract (precis)

The rapid confirmatory Bio-Rad Geenius HIV 1/2 assay was evaluated as an alternative to the HIV-1 Western blot (WB) confirmatory assay. A total of 370 retrospective samples collected from 356 patients were tested.

## INTRODUCTION

Accurate laboratory diagnosis of human immunodeficiency virus (HIV) infection is essential for identifying individuals who could benefit from antiretroviral therapy and for reducing the risk of HIV transmission. HIV prevalence is considered low in Canada, with an estimated rate of 212 per 100,000 people; among infected people, 21% are unaware of their infection (1). The estimated incidence rates in Canada and Quebec are 7.2 and 3.4, respectively, per 100,000 people (1, 2). The national guidelines for the diagnosis of HIV infection recommend the use of a two-step algorithm in which specimens are first tested using an HIV-1/2-sensitive screening assay followed by a more specific confirmatory assay, such as Western blot (WB) (3).

In the province of Quebec, HIV screening is performed using mainly fourth-generation enzyme immunoassays (EIA) or chemiluminescence microparticle immunoassays (CMIA) (4).

Per the HIV diagnostic algorithm of Quebec, specimens with repeatedly reactive EIA/CMIA results undergo confirmatory testing at the Public Health Laboratory of Quebec. Since 1993, the HIV-1 WB assay (Bio-Rad Laboratories, Redmond, WA, USA) has been used as a confirmatory test to detect the presence of anti-HIV-1 IgG (5). Specimens with negative or indeterminate results by the WB are submitted to supplemental confirmatory assays using HIV-1 p24 antigen EIA (bioMérieux, Marcy- l’Étoile, France), which detects acute/early infection, and/or Inno-Lia HIV-1/HIV-2 (Fujirebio, Ghent, Belgium), which identifies both HIV-1 and HIV-2 infections.

As the third- and fourth-generation EIAs and the WB are not able to discriminate between HIV-1 and HIV-2, misclassification of HIV infection can occur (6). Such misclassifications lead to indeterminate or negative results (7, 8). Although the Inno-Lia confirmatory test is able to differentiate between HIV-1 and HIV-2 infections, it is time-consuming and provides subjective interpretation of results. This assay is not approved for HIV diagnosis by Health Canada.

In October 2015, Health Canada approved the Geenius HIV 1/2 confirmatory assay (Bio-Rad Laboratories, Marnes-la-Coquette, France) to confirm HIV infections. This single-use immunochromatographic assay, which uses recombinant and specific synthetic peptides, provides an appreciable turnaround time below 30 min, and the results are read and interpreted by a proprietary reader with dedicated software (9).

Our study aimed to evaluate the performance of the Bio-Rad Geenius HIV 1/2 assay as an alternative to HIV-1 WB in confirming HIV-1 and HIV-2 infections in both living patients and cadaveric blood samples.

## MATERIALS AND METHODS

### Clinical samples.

A total of 370 retrospective diagnostic samples collected from 356 patients were obtained from the Laboratoire de Santé Publique du Québec (LSPQ) serobank collection. The LSPQ is the Public Health Laboratory of the Province of Quebec, where confirmation of HIV infection is performed. All specimens used in this study are residual samples collected for routine diagnostic purposes. All samples were repeatedly reactive using a screening immunoassay, such as Architect from Abbott Laboratories or GS HIV-1/HIV-2 Plus O EIA from Bio-Rad Laboratories (HIV-1/HIV-2 Plus O EIA). Screening immunoassays were performed according to the manufacturer’s instructions. Samples included 7 panels. Panel 1 had 57 HIV-1/HIV-2-negative serum samples obtained from individuals with no documented HIV infection; all were negative using a screening immunoassay. Panel 2 had 58 HIV-1-positive serum samples, including 38 HIV-1 subtype B serum samples and 20 HIV-1 non-subtype B serum samples. Among the 38 HIV-1 subtype B serum samples, 20 were from recently HIV-1-infected patients that were positive by WB (10), 10 were from chronically HIV-1-infected patients also positive by WB, and 8 were from HIV-1-infected patients that were indeterminate by WB and positive by Inno-Lia. The 20 HIV-1 non-subtype B samples included 6 C, 3 CRF02_AG, 3 D, 2 A1, 2 CRF01_AE, 2 CRF06_CPX, and 2 G samples. The selection of these samples was determined based on the distribution of the HIV-1 non-subtype B samples in Québec. Panel 3 had 36 HIV-2-positive serum samples obtained from individuals with documented HIV-2 infection, confirmed by radioimmunoprecipitation assay (RIPA), Inno-Lia, or both. Panel 4 had 9 HIV-infected patients presenting with a positive but untypeable HIV result by Inno-Lia and an indeterminate result by WB. Panel 5 had 110 serum samples collected between 2012 and 2015 from individuals presenting with repeated indeterminate results using both WB and Inno-Lia. Panel 6 had 20 serum samples obtained from 10 seroconverted HIV patients. For these patients, the 10 first-drawn samples were all positive by screening immunoassay, 6 of them were indeterminate, and 4 were negative by WB and Inno-Lia confirmatory assays. In addition, all 10 first-draw specimens were tested for p24 antigen, and 7/10 were confirmed positive for the presence of p24 antigen. The 10 samples drawn 1 to 2 weeks later were positive by a screening immunoassay and by WB. Panel 7 had 80 cadaveric samples, including 54 obtained from patients with no documented HIV infection that were negative by a screening immunoassay, 23 HIV-1 positives obtained from individuals with a documented HIV-1 infection that were positive by WB, and 3 samples obtained from individuals presenting with repeated indeterminate results using both WB and Inno-Lia.

Patients were defined as HIV positive based upon repeat reactive HIV-1/HIV-2 Plus O EIA results and a positive WB. The three cadaveric samples considered indeterminate were positive using HIV-1/HIV-2 Plus O EIA and indeterminate using both WB and Inno-Lia.

### HIV assays.

All samples were tested with the Geenius HIV 1/2 confirmatory assay. Geenius is a single-use immunochromatographic test approved by Health Canada for the confirmation and differentiation of HIV-1 and HIV-2 in fingerstick whole-blood, venous whole-blood, serum, and plasma samples. Geenius employs antibody protein A conjugated to colloidal gold dye particles. Four HIV-1 antigens (Env gp160 and gp41, Pol p31, and Gag p24) and two HIV-2 antigens (Env gp140 and gp34) are bound to a nitrocellulose strip. gp160 and p24 are recombinant proteins, and the other antigens are synthetic peptides (11). Geenius was performed according to the manufacturer’s protocol (9). The protocol for venous whole blood was applied to cadaveric blood samples. Briefly, either 5 µl of the serum sample or 15 µl of the cadaveric blood sample was applied to the sample/buffer well. After migration of the sample onto the strip, additional buffer was added and a 20-min incubation was applied. The assay is completed within 30 min, and results are read and interpreted by dedicated software on the Geenius system. Geenius is interpreted as positive for HIV-1 if two bands of the HIV-1 test lines with at least one Env protein (gp160 or gp41) are present. Positivity for HIV-2 is assumed if both HIV-2 bands are present.

The HIV-1 WB confirms the presence of antibodies to HIV-1. It was performed according to the manufacturer’s instructions (5). Briefly, individual nitrocellulose strips, containing HIV-1 proteins separated according to their molecular weights, are incubated with each specimen. The position of bands on the strips allows the antibody reactivity to be associated with specific viral antigens. The assay allows an evaluation within 3 h. The patients’ results were interpreted according to the Centre for Disease Control (CDC) and Health Canada interpretation criteria (12). A positive test result is defined by the presence of any two of the following bands: p24, gp41, and gp120/160.

Inno-Lia is a line immunoassay that detects antibodies against five HIV-1 antigens (Env gp120 and gp41, Pol p31 and p17, and core p24) and two HIV-2 antigens (Env gp105 and gp36). Patient specimens were incubated overnight with the test strips. Afterwards, unbound material was washed and an enzyme-labeled antibody was used to visualize the HIV antigen/antibody complexes. The assay provided a result within 20 h. The Inno-Lia was interpreted as positive when two lines are visible, one of which must be for Env.

HIV-1 p24 antigen detection and confirmation were performed using the automated VIDAS HIV p24 II detection and confirmation assays according to the manufacturer’s instructions.

Viral load quantification was obtained using the Abbott RealTime HIV-1 assay in plasma specimens. Testing was performed as described by the manufacturer.

### Statistical analysis.

We performed comparisons of sensibility, specificity, and indeterminate proportion between Geenius and WB using McNemar’s exact test for the comparison of two proportions on paired data. We performed statistical analysis using SAS 9.4 (SAS Institute). Statistical tests were two sided, with significance assigned at a *P *value of <0.05.

## RESULTS

The performance of the Geenius test in the confirmation of HIV infection in serum samples is shown in Table 1. Among the 57 HIV-negative samples (panel 1), 53 were correctly identified by Geenius compared to 51 by WB. Four specimens were indeterminate with Geenius and 6 with WB, leading to a specificity rate of 93% for Geenius and 90% for WB. None of the HIV-negative samples were positive by Geenius or WB.

**TABLE 1:** Performance characteristics of Geenius and Western blot by testing serum samples

HIV serum parameter	Geenius	WB	*P* value^*b*^
HIV negative panel (*n* = 57)			
No. correctly identified/total no.	53/57	51/57	
No. indeterminate/total no.	4/57	6/57	
Specificity (%)	93	90	0.6875
Indeterminate rate (%)	7	10	
HIV-1 positive panel (*n* = 58)			
No. correctly identified/total no.	58/58	50/58	
No. indeterminate/total no.	0/58	8/58	
Sensitivity (%)	100	86	NA
Indeterminate rate (%)	0	14	
HIV-2 positive panel (*n* = 36)			
No. correctly identified/total no.	32/36	NA	
No. untypeable/total no.	3/36	14/36	
No. indeterminate/total no.	1/36^*a*^	22/36	
Sensitivity (%)	97	39	<0.0001
Differentiation capacity (%)	89	NA	
Indeterminate rate (%)	3	61	
HIV Inno-Lia untypeable panel (*n* = 9)			
No. correctly identified HIV-1/total no.	7/9	NA	
No. correctly identified HIV-2/total no.	2/9	NA	
No. indeterminate/total no.	0/9	9/9	
Sensitivity (%)	100	NA	
WB/Inno-Lia indeterminate status (*n* = 110) (no./total no.)			
HIV-1	11^*a*^/110	NA	
Indeterminate	13/110	110/110	NA
Negative	86/110	NA	

a Patients presenting with discordant results (described in Table 2).

b McNemar's exact test may not be performed when one of the two assays presents only one result. NA, not applicable.

A total of 103 serum samples selected from 99 patients with known HIV infection status were tested. This group consisted of 58 HIV-1-positive (panel 2), 36 HIV-2-positive (panel 3), and 9 positive but untypeable HIV serum samples (panel 4). Sensitivity of Geenius for all 58 HIV-1-infected patients was 100% (58/58) compared to 86% (50/58) for WB. Geenius did not miss any HIV-1-positive patient samples. The sensitivity of Geenius to detect HIV-2 infection was 97% (35/36) compared to 39% (14/36) for WB.

Using panel 3, Geenius correctly identified 32 of the 36 HIV-2 infections, whereas 3 out of 36 specimens remained untypeable and one was indeterminate (Table 2, patient 12), leading to an HIV-2 differentiation rate of 89%.

**TABLE 2:** Investigation of discordant results between Geenius, WB, and Inno-Lia^*a*^

Patient no.	Specimen no.	Assay S/CO by generation		P24 Ag	Assay finding			
		3rd	4th		WB	Inno-Lia	Geenius	Comment(s)
1	1	24.80	216	Neg	Ind (gp160)	Ind (gp41)	HIV-1 pos (gp160, gp41)	Pos viral load (primary infection)
2	1	1.50	21.91	Neg	Ind (gp160, gp120)	Ind (gp41)	HIV-1 pos (gp160, gp41)	Pos viral load (primary infection)
3	1	15.60	35.50	Neg	Ind (gp160)	Ind (gp41)	HIV-1 pos (gp160, gp41)	HIV-1-pos known patient
4	1	1.56	42.90	Neg	Ind (gp160)	Ind (gp41)	HIV-1 pos (gp160, p31, gp41)	Pos viral load (primary infection)
5	1	13.20	55.63	Neg	Ind (gp160)	Ind (gp41)	HIV-1 pos (gp160, gp41)	HIV-1 RNA not detected
6	1	13.11	459	Neg	Ind (p24)	Ind (gp41)	HIV-1 pos (gp160, gp41)	HIV-1-pos known patient
7	1	13.50	85.58	Neg	Ind (gp160)	Ind (gp41)	HIV-1 pos (gp160, gp41)	HIV-1-pos known patient
8	1	Neg	1.14	Neg	Ind (gp160)	Ind (gp41)	HIV-1 pos (gp160, gp41)	HIV-1 RNA not detected
9	1	14.20	7.04	Neg	Ind (gp41)	Ind (gp41)	HIV-1 pos (gp160, gp41)	HIV-1 RNA not detected
10	1	14.90	11.86	Neg	Ind (gp160)	Ind (gp41)	HIV-1 pos (gp160, gp41)	HIV-1 positive known patient
11	1	13.80	22.06	Neg	Ind (gp160)	Ind (gp41)	HIV-1 pos (gp160, gp41)	HIV-1-pos known patient
12	1			Neg	Ind (p65, p55, p40, p31, p24, p18)	HIV-2 pos (p24, p17, sgp105, gp36)	HIV-2 ind (gp160, gp41)	HIV-2 RNA not available
	2			Neg	Ind (p65, p55, p40, p31, p24, p18)	HIV-2 pos (p24, Spg105, gp36)	HIV-2 pos (gp36, gp41, gp140)	HIV-2 RNA not available
13	1			Neg	Ind (p24)	HIV-1 pos (gp41, p24)	Neg	HIV-1 RNA not available
	2			Neg	Ind (p24)	HIV-1 pos (gp41, p24)	Ind (gp41)	HIV-1 RNA not available
	3			Neg	Ind (p24)	HIV-1 pos (gp41, p24)	Ind (gp41)	HIV-1 RNA not available
	4		Neg		Ind (p24)	Ind (p24)	Neg	HIV-1 RNA not available

a Abbreviations: S/CO, signal to cutoff; Ag, antigen; Neg, negative; Ind, indeterminate; Pos, positive.

Among the 9 HIV-untypeable patients (panel 4), Geenius assay confirmed 7 as HIV-1 and 2 as HIV-2 infections.

The fifth panel consisted of 110 serum specimens presenting with repeated indeterminate results for both WB and Inno-Lia. Geenius results were negative for 86, indeterminate for 13, and HIV-1 positive for 11. The Geenius negative and indeterminate results for the 86 and 13 patients, respectively, were consistent with expected results, since these patients did not seroconvert and were still indeterminate by WB and Inno-Lia when follow-up specimens were tested. Regarding the 11 Geenius HIV-1-positive patients, a clinical and epidemiological investigation was performed and results are presented in Table 2 (patients 1 to 11). All 11 patients reacted to the Architect Combo assay, and 10/11 also reacted to the HIV-1/HIV-2 Plus O EIA. All 11 were negative for p24 antigen and were indeterminate using WB and Inno-Lia. For all 11 patients, Inno-Lia detected gp41, while Geenius detected two envelope antibodies, gp160 and gp41. Inno-Lia detected gp41 in all 11 patients, while Geenius detected two envelope antibodies, gp160 and gp41. Criteria of interpretation for the Inno-Lia assay are based on the detection of antibodies against HIV-1 sgp120, gp41, p31, and p17, as well as antibodies against HIV-2 gp36 and sgp105 (13). The fact that Inno-Lia does not detect gp160 might explain the indeterminate result using such an assay. Follow-up specimens and clinical investigation of patients 1 to 11 showed that patients 1, 2, and 4 presented with positive viral load in addition to a follow-up positive result with WB. Patients 3, 6, 7, 10, and 11 were known to be HIV positive and were under antiretroviral treatment. Finally, patients 5, 8, and 9 had a negative viral load and no history or evident risk for HIV exposure. Patient 5 is a 37-year-old male hospitalized for a pulmonary embolism, patient 8 is a 34-year-old pregnant and healthy woman with no risk for HIV, and patient 9 is a 9-year-old female who recently had malaria and who was diagnosed with a latent tuberculosis infection. Follow-up specimens collected from those 3 patients were indeterminate with Geenius (positive only for gp41) as well as with WB and Inno-Lia (positive for gp41) (data not shown). Our results confirm that among 110 patients presenting with one or two positive screening assays and indeterminate WB and Inno-Lia results, Geenius showed 3/110 (2.7%) false-positive results.

Table 2 shows two other patients with discordant results (patients 12 and 13). Patient 12 is a well-characterized HIV-2 chronically infected patient presenting with an Inno-Lia and RIPA HIV-2 positive result while showing indeterminate result by Geenius (gp36 and gp41). A follow-up serum sample from this patient revealed a positive result by Geenius (presence of gp36, gp41, and gp140 bands). Four serial samples were obtained from patient 13. Whereas the first three drawn samples were HIV-1 positive by Inno-Lia, the last sample showed an Inno-Lia indeterminate result. According to the actual HIV confirmatory algorithm, patient 13 was first diagnosed as positive for HIV-1 and then confirmed as falsely HIV-1 infected in the last specimen. Geenius assay confirmed from the first specimen that patient 13 was not HIV infected.

Another objective of this study was to determine the capacity of Geenius to detect acute infections. For this purpose, we analyzed 20 samples from 10 patients in their early phase of HIV infection (panel 6). All first-draw samples (10/10) screened positive and 4/10 were confirmed negative, while 6/10 were indeterminate using both WB and Inno-Lia. All 6 WB- and Inno-Lia-indeterminate and 1 of the 4 WB- and Inno-Lia-negative first-draw samples were positive for p24 antigen. Interestingly, among the first-draw samples, 2 WB/Inno-Lia-indeterminate and 1 WB/Inno-Lia-negative samples were positive by Geenius. All three were also positive for p24 antigen (data not shown). The 10 samples drawn 1 to 2 weeks after the first samples were screened positive and confirmed positive by WB (10/10) as well as by Geenius (10/10). Thus, Geenius detected an HIV-1 infection in the first blood sample of three patients presenting with indeterminate or negative results by WB and Inno-Lia assays.

Finally, the performance of Geenius in confirming HIV infection in 80 cadaveric blood samples (panel 7) was investigated and is presented in Table 3. The sensitivity of Geenius to detect HIV-1 infection in cadaveric samples was 100% (23/23). Even though the specificity was 95% (52/54), none of the negative samples were HIV positive. Finally, 3 samples that were screened positive with HIV-1/HIV-2 Plus O EIA and confirmed indeterminate by WB and Inno-Lia tested negative by Geenius.

**TABLE 3:** Performance characteristics of Geenius in confirming HIV-1 infection in cadaveric samples

HIV cadaveric sample parameter (panel 7)	Geenius result
HIV negative (*n* = 54)	
No. correctly identified/total no.	52/54
No. HIV-1 indeterminate/total no.	1/54
No. HIV-2 indeterminate/total no.	1/54
Specificity (%)	96
Indeterminate rate (%)	4
HIV positive (*n* = 23)	
No. correctly identified/total no.	23/23
Sensitivity (%)	100
HIV-1 indeterminate (*n* = 3) (no./total no.)	
Negative	3/3

## DISCUSSION

The World Health Organization (WHO), the CDC, the Association of Public Health Laboratories (APHL), and the Public Health Agency of Canada (PHAC) recommend a sequential strategy for HIV testing (3, 14–16). The WHO recommends a series of 3 different immunoassays that detect preferentially HIV-1 and HIV-2 antibody as well as HIV-1 p24 antigen. According to the CDC and APHL, testing starts with a sensitive immunoassay that detects HIV-1 and HIV-2 antibodies and p24 HIV-1 antigen, followed, if necessary, by a more specific immunoassay that differentiates HIV-1 from HIV-2 antibodies and then, if necessary, by an HIV-1 nucleic acid test (NAT). All CDC- and APHL-recommended assays are approved by the U.S. Food and Drug Administration (FDA). The Canadian HIV testing strategy is similar to that of the recommendations of the CDC and APHL. However, nucleic assays are not recommended, since they are not approved by Health Canada for HIV diagnosis. For more than 25 years, WB has been the only HIV confirmatory gold standard assay approved in Canada. In addition to the WB, Quebec uses an EIA that detects and confirms the presence of p24 antigen as a supplemental assay to the WB and as an alternative to the NAT.

WB is a very specific and reliable assay, but it is known to be laborious, time-consuming, and subjective. WB produces indeterminate results, frequently causing delayed reporting. In addition, WB only confirms HIV-1, leading to the misdiagnosis of HIV-2 infections.

This study evaluated the performance of the Geenius HIV 1/2 assay for both confirmation and differentiation of HIV-1 from HIV-2 antibodies. Geenius demonstrated significant advantages over WB in both confirming and excluding HIV infection. Its specificity of 93%, compared to 90% for WB, was equivalent to that of previously reported results (17, 18). The sensitivity of Geenius to confirm HIV-1 infection (100%), evaluated using a panel of serum samples that includes group B (2/3) and non-B (1/3) subtypes, was higher than that of WB (86%). In addition, the sensitivity of Geenius to confirm HIV-2 infection was determined at 97%, while its HIV-2 differentiation capacity was 89%. One patient (Table 2, patient 12) with well-characterized HIV-2 chronic infection and presenting with an Inno-Lia- and RIPA HIV-2-positive result was indeterminate by Geenius (gp36 and gp41). A follow-up serum sample from this patient revealed a positive result by Geenius (presence of gp36, gp41, and gp140 bands). Although the performance of Geenius was described to be superior to that of Inno-Lia (19), Geenius was less sensitive than Inno-Lia in detecting one serum sample from this HIV-2-infected patient. Such a discrepancy was previously described (20). Based on these data, maintaining the Inno-Lia assay as a supplemental confirmatory test in our HIV diagnostic algorithm may be recommended.

Our HIV-2 sensitivity results are comparable to those of a recent study that showed a capacity of differentiation of 94% using Geenius (19). Because cross-reactivity between HIV-1 and HIV-2 antibodies is commonly observed using WB, HIV-2 infection prevalence in Quebec may be underestimated using the WB confirmation algorithm, and this issue should be resolved with the introduction of Geenius.

Results obtained from the HIV-1- and HIV-2-positive samples indicate that Geenius provides an advantage over WB with less indeterminate results (0% for HIV-1 and 3% for HIV-2 compared to 12% for HIV-1 and 61% for HIV-2 by WB).

In a previous study, Montesinos et al. (18) obtained a sensitivity of 92% for Geenius, compared to 89% for WB. Using WB as a gold standard, Moon et al. (21) obtained a sensitivity of 100% using Geenius, suggesting that Geenius did not miss any WB-positive sample. Compared to other confirmatory assays, such as Chiron RIBA, Inno-Lia, or multisport rapid test, Geenius presents excellent performance and has many advantages (11, 17, 19, 22, 23), especially in terms of specificity (99 to 100%) and sensitivity (96 to 100%). To our knowledge, only one study concluded that Geenius was less sensitive and less specific than Inno-Lia, mainly due to its short time of incubation (20 min compared to overnight for Inno-Lia) (20).

The 9 patients that were HIV untypeable by Inno-Lia and indeterminate by WB were classified as either HIV-1 (*n* = 7) or HIV-2 (*n* = 2) positive by Geenius. Given the small number of patients and the selection bias (WB indeterminate), we must be cautious in the interpretation of these results.

Results obtained from patients with repeated indeterminate status demonstrate that Geenius has the ability to reduce the rate of indeterminate results (*n* = 13). In addition, Geenius showed a higher sensitivity than WB and Inno-Lia by classifying 11 out of 110 patients as HIV-1 positive by Geenius. Our investigation of these 11 discordant results demonstrated that 8 were true positives that were missed, probably because Inno-Lia does not detect antibody against gp160. However, we obtained 3 Geenius false-positive results (2.7%) among the 110 HIV-indeterminate patients. As part of the routine diagnosis of HIV in Quebec, an average of 330,000 specimens are tested yearly using mainly a 4th generation assay (screening assay). All reactive samples (nearly 2,400/year) are then submitted for confirmation using WB assay. Samples that are indeterminate with WB (nearly 250/year) are then tested using Inno-Lia. Patients that are indeterminate with both WB and Inno-Lia are not very common (average of 28 per year). Thus, by using Geenius as an alternative to WB and Inno-Lia, we predict fewer than 1 (0.75) false-positive case per year. To our knowledge, this is the first study that evaluated the performance of Geenius in classifying a significant number of specimens from patients who are indeterminate by both WB and Inno-Lia. Previous studies tested 5 to 27 indeterminate specimens and observed that Geenius gave a reduced number of indeterminate results and was more sensitive than WB and Inno-Lia in identifying HIV infections (19, 21, 24). Geenius was also more specific than Inno-Lia. Among the thirteen patients presenting with discordant results in our study, one patient (Table 2, patient 13) presenting with a first WB-indeterminate result was considered HIV-1 positive using the Inno-Lia assay as a confirmatory/supplemental assay. When tested by Geenius, this patient was considered negative from the first specimen tested. A clinical and epidemiological investigation showed that this patient had no clinical symptoms, no evident risk of exposure to HIV, an undetectable viral load, a CD4^+^ T-cell count of 1,043, and a CD4/CD8 ratio of 1.8. A fourth specimen was drawn, and this time it was indeterminate by both WB and Inno-Lia. This patient was considered positive for HIV-1 until the fourth specimen was drawn, confirming the Geenius-negative and the Inno-Lia falsely positive results. In a recent study, Tinguely et al. (20) described a similar result where one patient out of 136 negative blood donors was weakly positive by Inno-Lia and negative by Geenius.

The capacity of Geenius to detect acute infections was investigated using 20 samples from 10 patients in their early phase of HIV infection. Their first-drawn sample was either negative or indeterminate using both WB and Inno-Lia, while the second sample was confirmed positive by WB. Interestingly, Geenius detected an HIV-1 infection in the first blood sample of three patients presenting with indeterminate or negative results by WB and Inno-Lia assays. The three patients were also positive for p24 antigen (data not shown). Although the number of patients that we tested was limited, our data suggest the benefit of using Geenius to expedite the detection of acute HIV infections. Tuaillon et al. (11) investigated the ability of Geenius to discriminate between stages of HIV infection. Although Geenius was less suitable for distinguishing between acute and chronic infections, these authors also confirmed that it had a good performance for HIV confirmation during the early phase of infection. Rakovsky et al. (25) recommended the Xpert Qual HIV-1 RNA assay for patients with negative or indeterminate Geenius result. In Quebec, molecular RNA assays are not routinely introduced in the HIV diagnosis algorithm. However, an assay that detects and confirms the presence of HIV-1 p24 antigen has the benefit of confirming or denying HIV acute infection for samples negative or indeterminate by Geenius.

A previous study (26) showed that Geenius presents a promising performance in testing dried blood spots. However, to our knowledge there are no studies on the performance of Geenius in cadaveric blood samples. Even the manufacturer of Geenius (Bio-Rad Laboratories) does not make claims for cadaveric blood samples. They did not evaluate the performance of this assay in confirming HIV infection in cadaveric samples or request an approval by Health Canada for this purpose. We believe this study is the first to investigate a large number of specimens (80) and to demonstrate a good sensitivity (100%) and specificity (95%) in confirming HIV infection directly from cadaveric blood samples.

This study showed that the Bio-Rad Geenius HIV 1/2 rapid test exhibits better sensitivity to detect HIV-1 infections and better performance than WB to confirm and differentiate between HIV-1 and HIV-2 infections. In addition, the ability of Geenius to confirm HIV infection in cadaveric blood samples was demonstrated. The high sensitivity of this new confirmatory assay to detect early infections as well as to reduce the rate of indeterminate status indicates that Geenius is a good confirmatory test alternative to WB.
